# Advances in Novel Flame-Retardant Technologies for Fire-Safe Polymeric Materials

**DOI:** 10.3390/molecules29030573

**Published:** 2024-01-24

**Authors:** Xin Wang, Weiyi Xing, Gang Tang

**Affiliations:** 1 State Key Laboratory of Fire Science, University of Science and Technology of China, Hefei 230026, China; 2 School of Architecture and Civil Engineering, Anhui University of Technology, Ma’anshan 243002, China

## 1. Introduction

This Special Issue, titled “Advances in Novel Flame-Retardant Technologies for Fire-Safe Polymeric Materials”, aims to detail the recent advances in the design and preparation of novel flame retardants for use in fire-safe polymeric materials. Due to developments in science and technology, fire-safe polymeric materials are increasingly widely used in transportation, construction, aerospace, electronics, and electrical devices [1,2,3].

**Figure 1:**
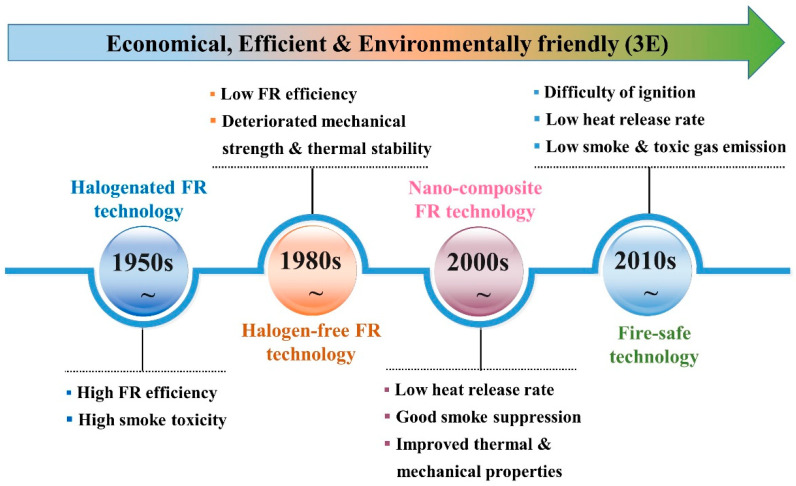
The development stages of flame-retardant polymeric materials.

The developments that have occurred in creating flame-retardant polymeric materials thus far can be roughly divided into four stages (Figure 1): (1) The early flame-retardant polymeric materials used halogenated flame retardants such as tetrabromobisphenol A. Despite their high efficiency as flame retardants, tetrabromobisphenol A generates toxic and corrosive gases such as hydrogen halide, which are harmful to human health and the environment [4]. Therefore, halogen-free flame-retardant polymeric materials have become the principal trend in recent years. (2) Halogen-free (mainly silicon-, nitrogen-, phosphorus-, and boron-containing) flame-retardant polymeric materials have emerged owing to their improved flame retardancy and environmental friendliness, but their mechanical strength and/or thermal stability usually deteriorate due to high loading. (3) Flame-retardant polymer nanocomposites exhibit the combined advantages of a low heat release rate, smoke toxicity suppression, and good mechanical and thermal properties. However, their level of flame retardancy struggles to meet industrial requirements such as a UL-94 V-0 rating. (4) The latest generation of flame-retardant polymers, also known as fire-safe polymer technology, not only achieves a UL-94 V-0 rating but also possesses a low heat release rate and low toxic smoke emissions.

Fire-safe polymers are difficult to ignite and have a low heat release rate and low smoke and toxic gas emissions. The fact that they are difficult to ignite reduces the probability of materials catching fire; a low heat release rate can suppress the flame spread rate and increase the amount of time personnel have to escape in the event of fire; and lower smoke and toxic gas emissions can reduce the number of casualties in fire accidents. Therefore, the application of fire-safe technology is a pertinent future development trend in polymeric materials.

This Special Issue contains seven original research contributions and four review articles related to fire-safe polymeric materials, including epoxy resins, rigid polyurethane foams, vinyl ester resins, and textiles.

## 2. An Overview of the Published Articles

In the Special Issue’s first contribution, Ma et al. synthesized a novel sulfur–phosphorus reactive flame retardant (SPMS) for use in epoxy resins (EPs). The co-addition of the SPMS and ammonium polyphosphate (APP) effectively promoted flame retardancy and smoke suppression in the EPs. Specifically, the peak heat release rate (pHRR), peak smoke production rate (pSPR), and total heat release (THR) of the EP containing 6.67% of the SPMS and 13.33% APP were 82.4%, 93.5%, and 61.4% lower than those of the pure EP, respectively. The results demonstrated that this sulfur–phosphorus flame retardant was highly effective at alleviating the fire risk in EPs.

Next, in the research paper by Korobeinichev et al. (contribution 2), the effect of a combination of 9,10-dihydro-9-oxa-10-phosphaphenanthrene-10-oxide-4,4′-diamino-diphenyl methane (DDM-DOPO) and graphene on the flammability of glass-fiber-reinforced epoxy composites (GFREPs) was investigated. The GFREP samples with flame-retardant additives showed a lower downward shift in the flame propagation rate than those without additives. Additionally, the measured thermal structure of the actual flames was compared with the results of numerical simulations of the flame propagation in the GFREPs.

Furthermore, in the study by Hu et al. (contribution 3), a layered ammonium vanadium oxalate-phosphate (AVOPh) was synthesized using a hydrothermal method. With the incorporation of 8 wt% of the AVOPh into the EP, the pHRR, total smoke production (TSP), and peak of CO production (PCOP) were 32.7%, 20.4%, and 37.1% lower than those of the pure EP, respectively. As a result, AVOPh was determined to be a promising high-efficiency flame retardant for use in EPs.

Chai et al. (contribution 4) synthesized a hybrid flame retardant (CuPPA-DOPO) by surface-grafting of amino phenyl copper phosphate with 9, 10-dihydro-9-oxygen-10-phospha-phenanthrene-10-oxide. With the addition of 6 wt% of the CuPPA-DOPO to the EP, the EP managed to reach the UL-94 V-1 classification and a limiting oxygen index (LOI) of 32.6%. In addition, compared with those of the pure EP, the pHRR and pSPR of the EP/6 wt% CuPPA-DOPO combination decreased by 52.5% and 26.1%, respectively. This favorable inhibition of the fire risk in the EP due to the CuPPA-DOPO was ascribed to the synergistic effects of the release of phosphorus free radicals during the gaseous phase and the catalytic charring ability of metal oxides during the condensed phase.

Meanwhile, in the research paper by Zhu et al. (contribution 5), a combination of steel slag (SS) and dimelamine pyrophosphate (DMPY) was used as a flame retardant for rigid polyurethane (RPUF). The pHRR and THR values of RPUF-3 when using the DMPY/SS system were reduced by 54.5% and 42.7% compared to when using unmodified RPUF, respectively. This study offers a novel strategy for the preparation of fire-safe RPUFs by utilizing metallurgical solid waste.

In the research paper by Xu et al. (contribution 6), a new phosphorus-based flame retardant, 6, 6′-(1-phenylethane-1,2 diyl) bis(dibenzo[c,e][1,2]oxaphosphinine 6-oxide) (PBDOO), was synthesized for use in vinyl ester resin (VE). The incorporation of 15 wt% of the PBDOO into the VE composites supported a UL-94 V-0 rating and a high LOI value of 31.5%. Additionally, the pHRR and THR of the VE containing 15 wt% of PBDOO were 76.71% and 40.63% lower than those of the unmodified VE, respectively. This study’s findings can be applied as an efficient approach to improving the fire safety of VE.

Kablov et al. (contribution 7) prepared elastomeric composites based on aluminosilicate microspheres, carbon microfibers, and a phosphor–nitrogen–organic modifier as part of their research, studying the composites’ fire- and heat-protective properties. The results showed that their linear burning speed was reduced by 6–17% compared to that of their known counterparts, which was attributed to the improved coke strength and catalyzed carbonization processes.

As regards literature reviews rather than novel studies on the topic, Yuan et al. (contribution 8) covered the recent progress in the field of flame retardancy and smoke suppression of RPUFs. Both conventional methods and innovative trends with respect to manufacturing fire-safe RPUFs, including reactive flame retardants, additive flame retardants, inorganic nanoparticles, and protective coatings, were analyzed in detail. Additionally, this review paper also proposed several challenges and future trends in this field.

Furthermore, Jin et al. (contribution 9) summarized the latest advances in multifunctional textiles, with a special focus on their flame-retardant and antibacterial properties. They describe how various treatment strategies, including the spray method, dip-coating, and pad-dry-cure methods; layer-by-layer (LBL) deposition; the sol-gel process; and chemical grafting modification, have been applied to endow textiles with multiple functions. Finally, the merits and drawbacks of these treatment strategies were compared, helping guiding future research and promoting the translation of the research into industry practice.

A review of the synthesis of supramolecular flame retardants (SFRs) based on non-covalent interactions was conducted by Xiang et al. (contribution 10). In this paper, first, different categories of SFRs of various dimensions were defined. Then, the influence of these SFRs on the fire-safe characteristics of typical polymers was emphasized. Additionally, the effects of the SFRs on the properties of polymeric materials, including their mechanical properties, were also evaluated.

In the final review paper, Feng et al. (contribution 11) explained the recent progress made in utilizing simple ball milling to fabricate flame retardants and flame-retardant polymer composites. First, they described how high-performance flame retardants were crushed, exfoliated, modified, and reacted using a ball mill. Then, they emphasized the incorporation of flame retardants into polymer composites using ball milling, with a special focus on the formation of multifunctional segregated structures. Their paper paves the way for simply and feasible developing fire-safe polymer materials.

## 3. Conclusions

Due to the increasing fire safety demands in the majority of their application contexts, polymeric materials need to be made flame-retardant. Consequently, a vast body of literature has become available on fire-safe polymeric materials over the past few decades. The emerging trend is undoubtedly toward eco-friendly, sustainable, and multi-functional technologies related to forming fire-safe polymeric materials [5,6]. Although many efficient flame retardants have been reported in the literature, transferring these newly arising fire-safe technologies from the research to an industry context will require further progress.
